# Workshop summaries from the 2015 Sex and Gender Medical Education Summit: utilization of sex and gender based medical education resources and creating student competencies

**DOI:** 10.1186/s13293-016-0092-8

**Published:** 2016-10-14

**Authors:** Alyson J. McGregor, Ana Núñez, Rebecca Barron, Robert Casanova, Eliza Lo Chin

**Affiliations:** 1 Division of Sex and Gender in Emergency Medicine, Department of Emergency Medicine, Warren Alpert Medical School of Brown University, Providence, RI USA; 2 Department of Medicine, Drexel University College of General Medicine, Philadelphia, PA USA; 3 Department of Emergency Medicine, Warren Alpert Medical School of Brown University, Providence, RI USA; 4 Texas Tech University Health Sciences Center, Lubbock, TX USA; 5 University of California, San Francisco, CA USA

## Abstract

**Background:**

Despite overwhelming evidence that sex and gender are critical factors in the delivery and practice of medicine, there is no unified sex- and gender-based medicine (SGBM) undergraduate medical education curriculum. Two Workshops within the 2015 Sex and Gender Medical Education Summit: a Roadmap to Curricular Innovation sought to lay the framework for such a curriculum.

**Methods:**

Attendees to the Sex and Gender Educational Summit self-selected attendance for one of two Workshops: (A) Utilization of SGBM Resources in U.S. Medical Schools or (B) Creating SGBM Student Competencies.

**Results:**

Workshop A identified gaps in existing curricula as well as strategies for incorporating available SGBM content into existing educational activities or curricular threads. Focus was given to the use of advisory committees to nurture collaboration and sharing of resources. Workshop B created a framework for national SGBM competencies by adapting existing materials from women’s health curricula such as Brown University’s SGBM Emergency Medicine subspecialty. The importance of student engagement, assessment, and faculty development were stressed as well as engaging the Liaison Committee on Medical Education (LCME) in awareness of the vital nature of including SGBM content into all medical school curricula.

**Conclusion:**

These Workshops provided a forum for national and international institutional representatives to lay a foundation for integration of SGBM into medical school curricula and the development of national SGBM Student Competencies.

**Electronic supplementary material:**

The online version of this article (doi:10.1186/s13293-016-0092-8) contains supplementary material, which is available to authorized users.

## Background

Despite the overwhelming evidence that sex and gender matter in health and disease and the delivery and practice of medicine, relatively little progress has been made to develop a unified sex- and gender-based medicine (SGBM) curriculum upon which future physicians can be educated. On October 18–19, 2015, the first “Sex and Gender Medical Education Summit: A Roadmap for Curricular Innovation,” was held at the Mayo Clinic in Rochester, Minnesota for faculty and students from more than 100 US medical schools, as well as representatives from European and Canadian institutions, federal agencies, and nonprofit organizations. This Summit was a collaborative initiative of the American Medical Women’s Association, the Laura W. Bush Institute for Women’s Health, the Mayo Clinic, and the Society for Women’s Health Research. During the Summit, national and international experts in medical school education discussed strategies for the integration of SGBM to ensure quality curricular development and assessment.

In order to successfully create national medical student competencies focusing on sex- and gender-based health, input from the conference attendees was sought through two corresponding Workshops: (A) Utilization of SGBM Resources in US Medical Schools: Overcoming Barriers to Achieve Action and (B) Creating SGBM Student Competencies in Alignment with The Association of American Medical Colleges (AAMC).” This summary describes the background for the Workshops and synthesizes the themes of topics presented and discussed.

## Workshop logistics

Participants were given the option to attend Workshop A or Workshop B prior to the Summit. Pre-work questions and reference readings were provided in advance and made available on the Summit website [1]. An expert faculty member in medical education or curriculum development was paired with an expert in sex- and gender-based medical evidence to serve as “Facilitator” and “Co-Facilitator” and to guide discussions for each small group. A scribe was assigned as notetaker and attendance recorder (Additional file 1). There were 59 participants subdivided into five individual groups for Workshop A and 52 participants subdivided into three individual groups for Workshop B.

Each group met for 1 h and then reconvened where a representative from each group shared the main points of discussion. Consensus statements, transcripts, and summary reports from each Workshop were collated for the purposes of this manuscript and to further the goals of the Summit.

## Workshop A: Utilization of SGBM Resources in US Medical Schools

In this Workshop, participants shared information regarding examples of successful models of curricular integration for topics of SGBM at their institutions. Participants were given access to Workshop A Pre-work Assignment in Table 1. Key concepts collated from this Workshop include the following:

**Table 1:** Workshop A Prework Assignment

Pre-work questions	1. Consider an educational project that you initiated that was not successful. Why do you think it was not successful? 2. Describe an educational project that was successful. Why was it successful and how did you sustain it? 3. What strategies would you use to incorporate sex and gender based medical education at your institution?
Reference reading	1. “Advancing sex and gender competency in medicine: sex & gender women’s health collaborative” [8]

### Curricular threads

Effective curricular design should concentrate not only on critical content but also on integrated themes that build in complexity throughout the duration of the educational process. This integrated approach is referred to as “curricular threads”. Medical school curriculum developers, perhaps through the formation of an advisory committee, should define SGBM learning objectives, recommend strategies for integration of SGBM material into existing curricula, and ensure such material is in line with existing learning objectives, Liaison Committee on Medical Education (LCME) standards, and AAMC domains. Incorporation of SGBM into existing curricula as curricular threads holds promise as a means of disseminating and contextualizing this material. As curricular threads, SGBM, content area that does not fall along traditional disciplinary lines, would consistently and longitudinally complement existing content areas presented by faculty who understand the overall curriculum and make conceptual links between new and previously learned material. For example, a SGBM preclinical elective was established at the Alpert Medical School of Brown University, a semester-long course for first-year medical students that explores a variety of SGBM topics, with efforts to closely align with material simultaneously covered in the medical school curriculum to reinforce relevant knowledge.

### Faculty development

The role of faculty development cannot be overlooked. Faculty should be educated on concepts of SGBM and ways to incorporate these concepts into medical school curricula through a multifaceted approach. In particular, tailoring the presentation of SGBM to the needs of specific audiences and limiting criticism of individual faculty’s existing educational materials would be crucial to success. For example, clinical case presentations could involve patients of both sexes, compelling students to examine sex differences in patient presentation, diagnosis, and treatment. Importantly, faculty needs to be rewarded for the time and effort they invest in strengthening SGBM curricula.

### Barriers to implementation

A number of barriers to the incorporation of SGBM content into existing medical school curricula were identified. These include limited resources (e.g., faculty time and effort), time constraints within existing curricula, uncertainty regarding the ideal time to introduce this content to medical students, and persistent knowledge gaps in this area. An overarching theme among the groups is the concern for the present lack of knowledge and need for assessment of how much and in what format does SGBM content already exist within medical school curricula currently. This would require further clarification in order for additional and appropriate incorporation of SGBM content to be successful.

### Overall strategies for incorporation

One unique asset of SGBM is that it crosses all disciplines and health professions. In order to strengthen the potential for interdisciplinary relationships and maximize their benefits, it is essential to have the involvement of SGBM experts, the development of forums for interaction among key players, and sharing of existing SGBM resources.

Potential strategies to increase the incorporation of SGBM content are presented in Table 2. The importance of student involvement in this process should not be overlooked; students can be the force that drives faculty to include SGBM into the material they teach, spearhead the assessment of existing SGBM content, and even develop supplementary content. Program evaluation to determine the success and sustainability of any efforts to incorporate SGBM would be key. Ongoing surveillance of any clinical issues that arise as a result of increasing evidence of sex and gender differences would be useful going forward.

**Table 2:** Overall strategies for incorporating SGBM into undergraduate medical education

Developmental stage	Strategies	Examples
Planning	Raise awareness	SGBM Education Summit [1]
	Identify stakeholders and formulate a SGBM advisory committee	Source experts, educational experts, certifying and assessment agencies, medical school deans, faculty, students
	Assess curricular need	Needs assessment survey
	Collaborate with core faculty	Medical education deans and directors, curriculum development faculty of preclinical and clinical instruction, course leaders
	Review existing curricula for adaptation components	Alpert Medical School of Brown University [9] Texas Tech University Health Science Center [10] Charité Hospital (Germany) [11] Karolinska Institutet (Sweden) [12] The Institute of Gender and Health (Canada) [13] The University of Toronto’s Collaborative Graduate Program in Women’s Health (Canada) [14]
Implementation	Integrate resources into existing educational activities	Texas Tech University SGBM Curriculum: clinical cases, slide library, and learning modules [10]. PubMed Search Tool [15]
	Faculty development	Texas Tech University Laura W. Bush Institute for Women’s Health “Y does X make a difference” SGBM continuing medical education [16], collaborative planning and implementation of skills development and team-building among faculty, development of a train-the-trainer approach.
	National organizations	Liaison Committee on Medical Education Association of American Medical Colleges Sex and Gender Women’s Health Collaborative American Medical Women’s Association
	Student involvement	Identify student leaders to join SGBM Advisory Committee
	Develop shared materials	Online modules, educational portfolios, premade lecture slides, smartphone technology
Evaluation	Student assessment	Incorporate into existing student assessments of written exams, participation in problem-based learning small group sessions, and core clerkship performance-based methods
	Program evaluation	Interprofessional education and patient-centered outcome measurement. Consider W.K. Kellogg Foundation’s Logic Model [17]

## Workshop B: Creating SGBM Student Competencies in Alignment with the AAMC

In this Workshop, participants created a framework for the development of national SGBM medical student competencies. Participants were given access to Workshop B Pre-work Assignment in Table 3.

**Table 3:** Workshop B Prework Assignment

Prework questions	1. Define competencies, milestones and entrustable professional activities (EPAs). 2. How should we approach sex- and gender-based competencies for medical education? Use existing women’s health competencies as a model? Or develop a unique approach? 3. How should sex- and gender-based competencies be formulated?
Reference reading	1. “Competencies, milestones, and entrustable professional activities: what they are, what they could be” [18] 2. Foundations for a novel emergency medicine subspecialty: sex, gender, and women’s health [9] 3. Women’s Health Competencies—links to NAWHME and APGO [19, 20]

### Clarity of definitions and terminology

Broad concepts such as “one size does not fit all” were clear; however, the definition of sex and gender and its parameters are less clear among educators. The phrase “every cell has a sex, every person has a gender” from the Institute of Gender and Health, Canadian Institutes of Health Research, was viewed as a useful awareness raising statement. Clarifying these terms and raising awareness is an important first step in developing effective, achievable competencies. Creating the umbrella definition of sex and gender biology in medicine and identifying the subset that encompasses the spectrum of sexuality and gender expression will be important in assisting faculty and students as they frame SGBM competencies.

### Educational philosophy

There was broad consensus that SGBM should be inclusive versus “stand-alone” and serve as a “lens” for viewing data. This means inclusion of health of women and men, the Lesbian, Gay, Bisexual, Transgender (LGBT) community as well as a gendered lens to health. There was consensus that this inclusivity would broaden the support of the curricular adoption. Science and optimally, evidence-based data, needs to drive SGBM curricular content and competencies. Likewise, critical thinking using a SGBM lens (e.g., “did the study investigate sex differences and did they find anything of significance?”) needs to occur within both basic and clinical science. Since both biologic factors and social determinants of health often cluster within a patient, questions like “what is the influence of sex and gender in this scenario?” enhances the information that could guide patient care.

### Approach to curriculum and competency development

The initial dialogue centered on what approach would be most successful: the development of a unique set of competencies versus adapting existing women’s health competencies to meet the needs of SGBM (Table 4). Recommendations were given to follow a similar framework as the article “Foundations for a Novel Emergency Medicine Subspecialty: Sex, Gender and Women’s Health,” which entailed an initial needs assessment, review of existing curricula, informational interviews with content experts, and developing educational objectives. Due to the fact that SGBM curricula should encompass all health conditions, topics traditionally taught under the rubric of obstetrics/gynecology should also be included such as sexually transmitted infections and intimate partner violence. Additionally, establishing a panel of experts and using as a guide competencies from the World Health Organization [2], American College of Clinical Pharmacy [3], and the AAMC’s “Implementing Curricular and Institutional Climate Changes to Improve Health Care for Individuals Who Are LGBT, Gender Nonconforming, or Born with DSD: A resource for Medical Educations” [4] would be useful starting points.

**Table 4:** Source competencies for adaptation to sex- and gender-based medicine

1.	Women’s Health in the Medical School Curriculum: report of a survey and recommendations. Advanced Curriculum on Women’s Health—American Medical Women’s Association (AMWA) [21]
2.	Women’s health care competencies for medical students—The Association of Professors of Gynecology and Obstetrics (APGO) [19]
3.	Women’s Health in the Curriculum: National Association of Women’s Health Medical Educators (NAWHME) [20]
4.	Council of Graduate Medical Education (CoGME) Fifth Report: Women & Medicine World Health Organization [2]
5.	Standards of Practice for Clinical Pharmacists. Pharmacotherapy American College of Clinical Pharmacy [22]
6.	Implementing curricular and institutional climate changes to improve health care for individuals who are LGBT, gender nonconforming, or born with differences in sexual development: a resource for medical educations—Association of American Medical Colleges (AAMC) [4]

### Framework for growth

There was consensus that sex- and gender-based differences should be integrated into curricula from the very beginning of medical school to ensure that students understand the basic science that supports these concepts. Numerous connections to integral elements of current curricula were proposed. These elements include evidence-based medicine, precision medicine, professionalism, patient-centered care, sociocultural determinants of care, sex as a biologic variable, healthcare delivery, and the “Triple Aim” of the Institute for Healthcare Improvement (improving quality and satisfaction, population health, and decreasing per capita cost). Integration within standard lectures, facilitated small group discussions, Objective Structured Clinical Examinations, standardized patients, and clinical experiences are all educational opportunities to facilitate understanding of SGBM. Case-based scenarios can be conducted by asking students to alter a given scenario by changing the sex or gender of the patient under discussion. SGBM may also be incorporated into entrustable professional activities (EPAs), which are professional activities based on specialty [5]. For example, incorporation of SGBM principles into the critical competency “consideration of cost awareness and risk-benefit analysis in patient care: Systems Based Practice (SBP3),” might include trainees’ ability to recognize lifelong gender pay inequities result in poverty of many elderly women [6]. As a result, trainees would routinely screen and inquire about a patient’s ability to “pay the bills” and obtain critical medications. This SGBM-integrated skill is critical in achieving optimal treatment outcomes. Integration is achievable as EPAs are comprehensive and many include components of cultural sensitivity. Another example is EPA 10: recognize a patient requiring urgent or emergent care and initiate evaluation and management. Women presenting with heart disease might not have the classic symptom of chest pain, which is listed as one of the presenting symptoms in this EPA. Students should be taught that with respect to SGBM, women might present with different symptoms that are considered equivalent anginal symptoms and should prompt the same recognition of the need for urgent or emergent care.

The LCME will need to be made aware of the critical nature of including SGBM content, perhaps developing this as an LCME “hot topic.” LCME site visitors will eventually also need to undergo some level of education so that they are able to assess if inclusion of SGBM concepts at a given institution has been adequate and comprehensive. Additionally, collective work should occur to increase the number of SGBM test items on the United States Medical Licensing Examination.

### Engaging stakeholders

The two most cited and integral stakeholders were students as “curricular drivers” and faculty. Engaging students to become change agents is essential to attaining true integration. If students perceive that sex and gender were not addressed during a particular educational experience, they should be encouraged to inquire about it openly. Evaluations by students should be updated to include specific assessment of SGBM content within the curriculum and personal competence and comfort in this area.

As SGBM is a new area, faculty development will be crucial. Factors promoting curricular adoption included both incentives and disincentives in order to achieve needed outcomes. For example, having the Dean charge the curricular group with a mandate and then recognizing the group for its contributions is more likely to drive desired results. The curricular groups may include both faculty at the parent site, as well as faculty involved with preceptorships and other clinical experiences. Training in SGBM could be integrated into existing annual faculty development or competency assessments. Inclusion of SGBM should also be added to existing conflict of interest forms signed by visiting speakers, so that these speakers are aware that their audience will be making assessments on it.

### Goals of achievement

In addition to being evidence based, competencies must generate objectives that are measurable. Assessment needs to ensure that the topic is included and comprise measures that assess higher order objectives (e.g., integration, application, and synthesis). Dynamic and ongoing evaluation strategies need to be employed to assess competency achieved and gaps in focus.

Ultimately, inclusion of SGBM would continue to improve medical student’s understanding of precision medicine and patient-centered care as they learn to utilize a sex and gender lens when viewing data or evaluating patients. This would be consistent with recommendations from the recent American Medical Association ChangeMedEd 2015 conference, at which participants recommended that patient-centered care and social determinants of health be emphasized more often and earlier in the curriculum. The use of a theoretical framework in education (e.g., Miller’s pyramid of knowledge, knows how, shows how and does) can cover multiple competency levels and progressive achievement of milestones. For example, students can acquire information about sex differences in myocardial infarctions (“knowledge”). They progress to identifying sex differences in presentation (“knows how”), diagnosing an MI in a women with risk factors and unexplained fast heart beat (“shows how”) and ultimately, routinely screening women with heart disease risk factors and accurately identifying unusual chest pain, nausea, and profound fatigue as presentations of an MI (“does”). This framework serves another purpose. When deployed, it provides achievable “floors” that all schools can be held to account.

## Conclusions

The input of national leaders in both medical education and SGBM provided a forum to address both content and implementation strategies across a broad range of concerns. Examination of current curricular models helped define best practices as well as identify barriers to widespread implementation. Initial steps for integration of new knowledge could begin immediately through the use of online modules, premade lecture slides, and case studies while plans to ensure sustainability would include the development of electives or curricular threads. The need to involve key stakeholders, both students and faculty, was emphasized, as was the need to identify the current SGBM content currently being taught at each institution. The next steps for creating SGBM student competencies were outlined through a clarification of the nomenclature of sex and gender, discussions on approaches to competency development, and proposals for a framework to build on this work. An underlying theme was the need for a gender lens in viewing healthcare in order to understand the contribution of sex and gender as determinants of health. Ultimately, the development of SGBM student competencies will need to align with current LCME standards and be assessed on standardized tests.

In summary, Summit Workshops examined the current status of SGBM education across a broad spectrum of institutions throughout the USA and outlined a framework for advancing this work through integration of teaching tools and the development of formal SGBM student competencies. Materials presented during the Summit, along with an SGBM toolkit [7] and publication of the Summit proceedings [1], will provide resources for workshop participants to take back to their home institutions as they continue to advance this discussion.

## Additional file

Additional file 1: Appendix A. Summit workshop attendance. (DOCX 102 kb)

## Abbreviations

AAMC: Association of American Medical Colleges
AMWA: American Medical Women’s Association
EPA: Entrustable professional activities
LCME: Liaison Committee on Medical Education
LGBT: Lesbian, Gay, Bisexual, Transgender
SBP: System’s Based Practice
SGBM: Sex and gender based medicine
