# Supplementary material for: Workshop summaries from the 2015 Sex and Gender Medical Education Summit: utilization of sex and gender based medical education resources and creating student competencies
Source: Biol Sex Differ. 2016 Oct 14;7(Suppl 1):43. doi: 10.1186/s13293-016-0092-8 (PMC5073901; doi:10.1186/s13293-016-0092-8)
Supplement: Additional file 1: Appendix A. — Summit workshop attendance. (DOCX 102 kb) [file 13293_2016_92_MOESM1_ESM.docx]

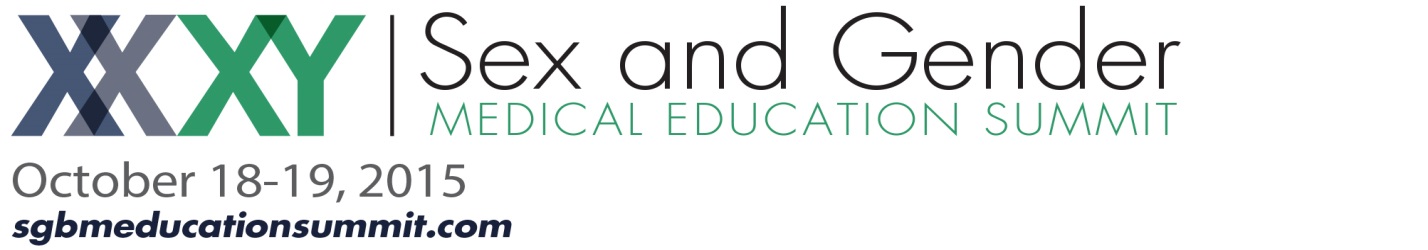


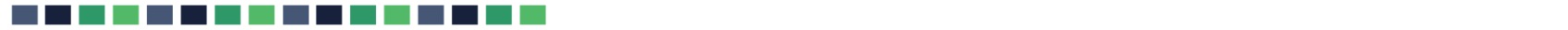


**Appendix A: Summit Workshop Attendance**

**Workshop A: Utilization of SGBM Resources in U.S. Medical Schools**

**Group A1**

**Facilitator**: Linda Solis

**Scribe**: Michelle Lyman

Anat Biegon

Rebekah Rollston

Aaron Marshall

Neelum Aggarwal

Jessica Servey

Sara Shields

Katie Leslie

Connie Tyne

Roberta Gebhard

**Group A2**

**Facilitator**: Suzanne Harrison

**Co-Facilitator**: Virginia Miller

**Scribe**: Alyssa Hermann

Nahid Azad

Herodotos Ellinas

Robert Bettiker

Patricia Robertson

Shivani Dhawan

Susan Bergeson

Sarah Blutt

Jane Balbo

Amy Stenson

**Group A3**

**Facilitator:** Patricia Garcia

**Co**-**Facilitator**: Ana Nunez

Jabbar Bennett

John Luk

Karen Reynolds

Theresa Rohr-Kirchgraber

Robin Jacobs

Judith Wolf

Gretchen Case

Nailah Cash-O’Bannon

Keisa Bennett

**Group A4**

**Facilitator**: Kahtrine Digre

**Co-Facilitator**: Alex Mechaber

**Scribe**: Facilitators

Elizabeth Mueller

Susan Hingle

Laryssa Kaufman

Kristin Dardano

Tanya Friese

Iman Mohamed

Henry Ng

Burton Ober

Caroline Davidge-Pitts

Mary Rojek

Alvaro Perez

Marcia Stefanick

Frederick Nagel

**Group A5**

**Facilitator**: Pamela Schaff

**Scribe**: Caroline Abercrombie

Carla Lupi

Cameron Crandall

Rita Lee

Jane McElroy

Deborah Vaughan

Kimberly Looney

Katie Imborek

Justin Sitron

**Group B1**

**Facilitator**: Robert Carroll

**Scribe**: Marley Hoggatt

Jennifer McCall-Hosenfeld

Anna-leila Williams

Jay Behel

Deborah Vozzella Hall

Jilyan Decker

Scott Moffatt

Hosanna Au

Gary Tithecott

Glenna Bett

Kathryn Spanknebel

Charlotte Reback

Mark Townsend

**Group B2**

**Facilitator**: Wendy Klein

**Co-Facilitator:** Jan Werbinski, Michelle Forcier

**Scribe**: Katie Baird, Rebecca Barron

Benoit Dube

Brandy Shattuck

David Waters

John Kazilionis

Rachel Brook

Karoline Kublickiene

Elizabeth Petty

Amy Bourns

Monica Cummings

Sharon Hull

Samia Osman

**Group B3**

**Facilitator**: Kim Templeton

**Co**-**Facilitator**: Michelle Berlin

Janet Pregler

Giulia Bonaminio

Eileen Burnett

Alyssa Rheingold

Sonya K Sobrian

Garren Gebhardt

Christine Peterson

Denise Kay

Juliana Kling

Nancy Weigle

Rania Cannaday

E. Lee Rosenthal

Sana Loue

Cynda Ann Johnson

Robert Ketchum

Lauren Walter

Robert Casanova

Sue Cox

Camilla Curren

Alyson McGregor

**Workshop B: Creating SGBM Student Competencies in Alignment with the AAMC**
